# Neonatal Markers of Prematurity as Predictors of Permanent Childhood Hearing Loss and Neurodevelopmental Impairment in Children Admitted to the Neonatal Intensive Care Unit

**DOI:** 10.3390/brainsci14090926

**Published:** 2024-09-17

**Authors:** Hayma Moosan, Derek J. Hoare, Dulip Jayasinghe, Karen R. Willis, Katherine Martin, Sally K. Thornton

**Affiliations:** 1 Hearing Sciences, Mental Health and Clinical Neurosciences, School of Medicine, The University of Nottingham, Nottingham NG1 5DU, UK; mzyhm22@exmail.nottingham.ac.uk (H.M.); sally.thornton@nottingham.ac.uk (S.K.T.); 2 NIHR Nottingham Biomedical Research Centre, Nottingham NG1 5DU, UK; 3 Neonatal Intensive Care Unit, City Hospital Campus, Nottingham University Hospitals, Nottingham NG7 7NW, UK; dulip.jayasinghe@nuh.nhs.uk; 4 The Children’s Audiology, Nottingham University Hospitals, Nottingham NG1 5DU, UK; 5 Child Development Centre, City Hospital Campus, Nottingham University Hospitals, Nottingham NG7 7NW, UK; katherine.martin3@nuh.nhs.uk

**Keywords:** hearing loss, prematurity, z-scores, high risk infants, neonatal intensive care unit, developmental outcomes, neurodevelopmental impairment

## Abstract

Need for admission to the neonatal intensive care unit (NICU) confers an increased risk of hearing loss in the newborn and of later neurodevelopmental impairment. In this retrospective longitudinal case-controlled study, we assess how the degree of prematurity, measured via gestational age, birth weight, and z-scores, in 138 infants admitted to the NICU are associated with permanent childhood hearing loss (PCHI) and 2-year developmental outcomes. Logistic regression analyses, Kruskal–Wallis analysis of variance, and Chi-squared tests were used. Independent of prematurity, PCHI and NICU admission were predictive of poor developmental outcomes. Twenty-one (47%) children with PCHI had a moderate-to-severe developmental delay, compared to three (7%) matched controls. Days in the NICU but not z-scores predicted PCHI. Z-score was not prognostic of moderate or severe developmental impairment in children with PCHI. The odds ratio of moderate-to-severe neurodevelopmental impairment with PCHI was high, at 12.48 [95% CI = 3.37–46.40]. Children with PCHI were significantly more likely to have cerebral palsy than their matched counterparts (30% vs. 2%). These findings challenge the conventional focus on gestational age and birth weight on neurodevelopmental outcomes for children with PCHI and NICU admission. A more nuanced approach to monitoring and intervention is needed.

## 1. Introduction

Permanent childhood hearing impairment (PCHI) is a common congenital anomaly and the fourth leading cause of disability globally. Prevalence estimates vary from 1–3 infants per 1000 and are approximately ten times higher in infants admitted to the neonatal intensive care unit (NICU) as a neonate [1,2,3]. Prematurity and admission to the NICU are predictors of both PCHI [4,5] and poorer neurodevelopmental outcomes [6,7,8]. Risk factors for PCHI are multiple, complex, and likely compounding [9,10,11,12,13,14,15,16]. The aetiology of PCHI can be diagnosed in about half of all cases [17,18]; a genetic cause is present in 50–60% of these infants, peripartum problems in 21%, and cytomegalovirus (CMV) infection in 19% of cases. In susceptible children, ototoxic medication (aminoglycosides) and diuretics (furosemide) are also risk factors for hearing loss [19,20]. Advancements in NICU medical technology and a proactive approach to resuscitation and intensive care of very preterm infants means that the viable gestational age has lowered, and survival rates have increased significantly in the last decade [21].

Few studies document associations between prematurity and PCHI. In a small case-control study (*N* = 93), six children (6.4%) of very low birth weight babies (<750 g) had sensorineural hearing loss [22]. Hearing loss was associated with ventilation, furosemide administration, and chronic lung disease. Hearing loss was also frequently associated with neurological comorbidities. A study (*N* = 67) assessing auditory evoked potentials at term in preterm infants (24–27 weeks) showed a higher risk of impairment of the brainstem auditory pathway and a higher frequency of PCHI than for infants born later (≥27 weeks), and the authors postulated likely causes were chronic lung disease and neurological morbidities [23]. In a larger preterm population, prevalence of hearing loss increased with decreasing gestational age and decreasing birth weight [24]. However, hearing loss was not necessarily permanent, as hearing loss was only identified at the screening stage and many false positives can be present at screening. Estimates vary from 7–24% of children who are referred via the hearing screening who then will have a permanent hearing loss (https://www.gov.uk/government/publications (accessed on 2 June 2024)) [25]. 

There is still contention in the literature as to whether prematurity and/or birth weight are significant risk factors for PCHI [4,5,16]. In a meta-analysis, prematurity and being small for gestational age (SGA) were not associated with PCHI [26]. However, most studies had a low or very low certainty of evidence. Factors for which there were moderate-certainty evidence were low birth weight, congenital anomalies, craniofacial anomalies, and intracranial haemorrhage [16]. From the available evidence, it is difficult to understand the relationship between prematurity and PCHI as the populations studied differ widely. Comparisons between studies can be problematic, as they may include only very preterm infants, also include well-baby populations, or those babies referred based on newborn hearing screening rather than with a later confirmed PCHI [24]. To determine relationships between prematurity, PCHI, and neurodevelopmental outcomes, it is important to include a control group matched on prematurity (F, gestational age, and weight) to elucidate factors prognostic of PCHI or later developmental outcomes independent of prematurity. 

It is also important to detect PCHI early as there are negative consequences associated with undetected or late detected PCHI, such as speech, language, and cognitive delays, poorer social adjustment, and lower educational attainments [27,28,29]. There is consensus in the hearing research community that earlier diagnoses, early treatment (hearing aids or cochlear implantation) and follow-up of PCHI in children are important for them to achieve satisfactory developmental outcomes [29,30,31]. Both physical and psychological co-morbidities can co-exist with PCHI [32] and it is thought that more than a quarter of children with PCHI have additional needs [33,34]. A meta-analysis of the neurodevelopmental impairment, including hearing loss, at ages 4–8 years of children born at 22–25 weeks showed a statistically significant increase in moderate–to-severe neurodevelopmental impairment for each decreasing week of gestation in extremely preterm infants [35]. A systematic review of cognitive deficits and behavioural data in preterm infants found a 10–40% increased risk of cognitive problems and 25% higher risk of language impairments, and that 8–75% have poorer overall school performance [36]. 

Infants admitted to the NICU in the UK as neonates are eligible for enhanced developmental surveillance if they meet certain criteria (NICE guidelines NG72, 2023). Enhanced developmental support and surveillance by a multidisciplinary team is provided up to 2 years (corrected age) for children born preterm who have or are at risk of a developmental problem if they were born before 30 + 0 weeks’ gestation. They may also be followed up if they are born between 30 + 0 and 36 + 6 weeks’ gestation if they have a brain lesion on neuroimaging likely to be associated with developmental problems or disorders (grade 3 or 4 intraventricular haemorrhage or cystic periventricular leukomalacia) grade 2 or 3 hypoxic ischaemic encephalopathy, neonatal bacterial meningitis, or herpes simplex encephalitis in the neonatal period. Local guidelines (Risk of adverse developmental outcomes; https://nuhp.koha-ptfs.co.uk (accessed on 31 January 2024)) can differ from NICE criteria and children < 31 weeks or 31 weeks with one or more risk factors needing longer term neurodevelopmental follow up have access to enhanced support (in terms of additional advice and interventions). The aim is to support them after discharge from hospital, respond to their concerns, and provide surveillance for and hence reduce the impact of any developmental problems and disorders (NICE guidelines NG72). Additionally, local guidance indicates all children born before 28 + 0 weeks’ gestation receive a face-to-face developmental assessment at 4 years (uncorrected).

The current study is unique as it is case-controlled and assesses the neurodevelopmental impairment in 2-year-old children with PCHI and an admission to the NICU at birth, where we assess the association between PCHI and neurodevelopmental outcome and neonatal measures of prematurity (gestational age, birth weight, z-scores, and centiles). Our objectives were to (1) determine whether neurodevelopmental outcomes are significantly poorer in children with PCHI compared with matched controls, and (2) determine whether the degree of prematurity is prognostic of PCHI and neurodevelopmental outcomes in this cohort.

## 2. Materials and Methods

Data were collected as part of a service evaluation (registered with the institutional clinical effectiveness department: -23-674C) to assess the number of children with PCHI and a NICU admission who fulfilled the surveillance criteria for follow-up. This study used data from the Nottingham NICU research database (NEAT) under ethical approval for secondary analysis of routinely collected data (REC project ID: 292263), South Central Berkshire Research Ethics Committee. 

### 2.1. Description of Cases

The study population consisted of 14,037 babies born in Nottingham and admitted to the NICU in Nottingham between 1 March 2008 and 29 February 2020—0.3% of this population had BHL. The cases (*n* = 46) were babies admitted to the NICU in this same epoch identified via the newborn hearing screening programme (NHSP) and then followed up to confirm PCHI. When PCHI is cited in reference to the data, this refers to confirmed bilateral hearing loss. Degree of hearing loss was categorised according to the British Society of Audiology guidelines as mild, moderate, severe, or profound, where hearing threshold is measured in decibels of hearing level (dB HL) and is averaged over the frequencies 0.5, 1, 2, and 4 kHz for the better-hearing ear (https://www.thebsa.org.uk/guidance-and-resources/current-guidance/?subject=paediatric-audiology (accessed on 31 January 2024)). Hearing threshold data were taken from their most recent audiological report from a paediatric audiologist. Depending on age and stage of development, hearing data were taken from the available pure-tone audiogram or play and visual reinforcement audiometry. Hearing data were also extracted from air and bone conduction tests (diagnostic ABR—Biologic Navigator Pro or Medelec Synergy). Eight children had mild bilateral hearing loss (BHL) (20–40 dB HL), 24 had moderate BHL (41–70 dB HL), four had severe BHL (71–95 dB HL), and eight had profound BHL (>95 dB HL). Data for children with UHL from this database have been reported previously [32].

Data were also collected on two control groups of NICU infants. A matched control group of infants (*n* = 46) were matched individually with the 46 PCHI cases on gestational age (±1 week), birth weight (±100 g), sex, and whether they were in the NICU for ≤ or ≥48 h. The matched control group of infants passed the NHSP and had no known acquired PCHI at the time of data collection. Infants were matched on these factors as we wanted to understand the impact of PCHI independent of birth weight and gestational age. 

We also collected data on 46 peer-matched control infants. Peer-matched infants were admitted to the NICU and had no confirmed PCHI at the time of data collection, and they were matched with the cases only on city hospital location, birth date, and sex. We collected peer-matched group data as we wanted to compare the indices of prematurity between cases and a representative cohort of children admitted to the NICU with no hearing loss. 

We collected the birth characteristics and 2-year developmental outcomes from routinely recorded data in the neonatal clinical database Badgernet (Clevermed, Edinburgh), from paper notes, and from other local clinical databases. 

### 2.2. Inclusion Criteria

Inborn neonates admitted on the day of birth to Nottingham NICU were considered. The PCHI cases had to have permanent bilateral hearing loss (>20 dB HL in both ears) and to have been referred on the NHSP, and the PCHI confirmed with later audiological testing by a paediatric audiologist. 

### 2.3. Exclusion Criteria

Matched controls and peer-matched control cases were excluded if they were referred via the NHSP or if they later went on to have an acquired hearing loss (at the time of data collection). Cases were also excluded if their hearing screening was conducted out of region. Two infants in the cases with PCHI were deceased after discharge from the NICU, so the follow-up data for those infants were not available to be included.

### 2.4. Data Entry and Items 

Data entry onto NICU Badgernet database was completed by a member of the NICU medical team. Most 2-year developmental outcome data were extracted from paper notes that had been entered by Paediatric Consultants who are specialists in developmental follow-up. Children were diagnosed with developmental impairment if their developmental skills fell two standard deviations or more below the population mean in two or more developmental domains. In this study, infants were categorised as having developmental impairment if it was stated in their medical notes that they had a global developmental delay, fine motor delay, delay in communication, or developmental impairment. Degree of developmental delay was scored using the developmental quotient (developmental age/chronological age ×100). Infants were categorised as having normal development if this was recorded in their medical notes or was presumed if there were no referrals for treatment or support recorded for their developmental needs. Being small for gestational age (SGA) is defined as a birth weight of less than the 10th percentile for gestational age and sex. Z-scores were calculated from birth weight, gestational age, and sex using an online calculator (http://intergrowth21.ndog.ox.ac.uk/en (accessed on 31 January 2024)).

### 2.5. Statistical Analyses

A power calculation was not performed as the sample was opportunistic and the upper number limited by the number of PCHI cases with NICU admission at the Nottingham University Hospitals. Data were analysed in SPSS version 28.0 (SPSS Inc., Chicago, IL, USA). Data were normally distributed but skewed sufficiently to indicate non-parametric statistical tests should be used (Shapiro–Wilks test; *p* value < 0.05 indicative of statistical significance). Kruskal–Wallis tests were used for non-parametric between group analysis of variance and Mann–Whitney U tests for post-hoc between-group comparisons. For all statistical tests completed, a *p*-value of <0.05 was assumed to be statistically significant. Pearson’s Chi-squared test was the statistical test applied to categorical data. In subgroup analyses, neonatal factors and neurodevelopmental outcome data for cases with PCHI were compared with matched controls (Chi-squared test). The data for gestational age and birth weight were compared between cases and peer-controls since the matched control group had the same (matched) indices of prematurity (Mann–Whitney U test). Logistic regression analyses were used to determine which variables could predict neurodevelopmental outcomes and PCHI in the model. 

Z-scores based on gestational age, birth weight, and sex are commonly used for research purposes to assess how the growth data differs from population average data. Z-scores are also useful as a proxy for growth as gestational age, birth weight, and sex are used to calculate the z-score. Statistically, it is useful as both gestational age and birth weight cannot be put into the regression model as they have high multicollinearity, which can lead to unstable coefficient estimates.

## 3. Results

### 3.1. Demographics: Children with PCHI Were Significantly More Premature than Peer Controls and Spent More Time in the NICU

Data in Table 1 indicate that infants with PCHI weighed significantly less (PCHI median birth weight 1370 g; range 540–960 g versus; peer controls, median 2503 g; range 514–4220 g; U = 711; *p* = 0.007) and were born at a more premature gestational age (gestational age, 32 weeks; range 24–41 weeks vs. 36 weeks; range 25–42 weeks; U = 773; *p* = 0.026) than the peer-matched controls. They also had significantly longer length of stay in the NICU (median 44 days, range 1–152 days) compared with the peer-matched controls (median 8 days, range 2–141 days; U = 599; *p* = 0.001).

### 3.2. Time Spent in the NICU but Not Prematurity (z-Scores) Significantly Predicted PCHI

A logistic regression was run on the PCHI cases and peer controls to understand the effects of the z-score and time in the NICU on having a diagnosis of PCHI. Time in the NICU (B = 0.018, odds ratio = 1.018) statistically significantly predicted PCHI (χ^2^(2) = 12.55, Wald = 9.63, SE = 0.006, OR = 1.018; *p* = 0.002), but z-scores did not (OR = 0.826; Wald = 1.4; SE = 0.161, OR = 0.826; *p* = 0.237). The model correctly classified 70% of cases. 

### 3.3. Children with PCHI Were Not All Born Premature or Small for Gestational Age

**Figure 1:**
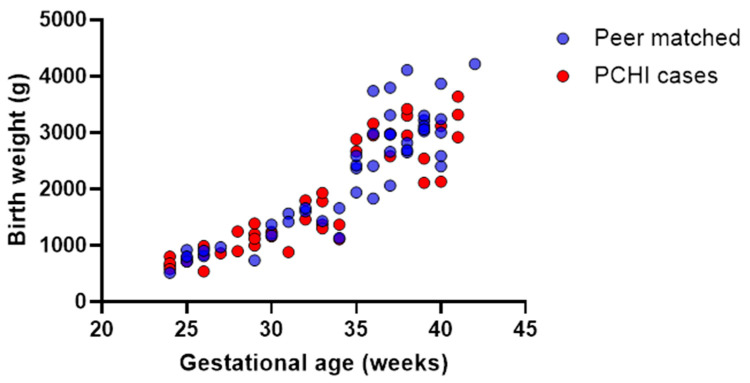
Full range of gestational ages and birth weights recorded for PCHI cases and peer-matched controls. Data from the cases with permanent childhood hearing impairment (PCHI) are shown as red symbols, peer-matched controls are shown as blue symbols. The peer-matched controls have also been admitted to the NICU and are matched on birth date, sex, and city location.

Gestational ages and birth weights were evenly distributed across all the birth weights and gestational age ranges in the PCHI group. This is exemplified in Figure 1, where birth weight is plotted against gestational ages for the cases with PCHI and the peer-matched controls. There were no disproportionate clusterings of cases with particularly lower gestational ages and birth weights for either group. This figure illustrates the strong positive correlation between birth weight and gestational age for children with PCHI and a NICU admission (Spearman’s correlation; r = 0.9; *p* < 0.0001) and for peer-matched controls (Spearman’s correlation; r = 0.8; *p* < 0.0001). The matched control group were not included in these analyses as the gestational ages and birth weights were matched (with the PCHI group).

### 3.4. Children with PCHI Were More Likely to Have Moderate or Severe Neurodevelopmental Delay

**Figure 2:**
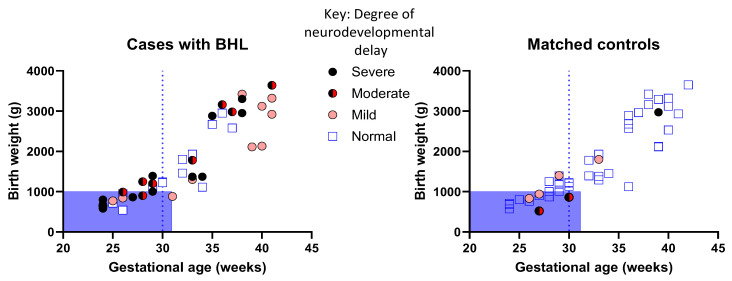
Children with PCHI were more likely to have moderate or severe developmental delay compared to matched controls and were not restricted to the most premature gestational ages. Blue shaded box indicates children who would be suitable for increased surveillance according to local guidelines. Dotted line indicates those children who would be followed up according to national NICE guidance. PCHI = permanent childhood hearing impairment. There was a strong positive correlation (assessed using Spearmen’s correlation) between birth weight and gestational age for cases with PCHI who have normal developmental delay (r^2^ = 0.77, *p* < 0.0001); mild developmental delay (r^2^ = 0.77; *p* = 0.0009); moderate developmental delay (r^2^ = 0.95, *p* < 0.001); and severe developmental delay (r^2^ = 0.82; *p* < 0.0001).

Figure 2 shows that for cases with PCHI and a NICU admission, a moderate or severe degree of developmental impairment was not, as might be assumed, restricted to the lower gestational ages and birth weights. In the matched control group, only three infants (7%) had moderate or severe developmental outcomes at 2 years of age.

### 3.5. Prematurity Is Not Prognostic of PCHI and Neurodevelopmental Outcomes in All Children with PCHI

**Figure 3:**
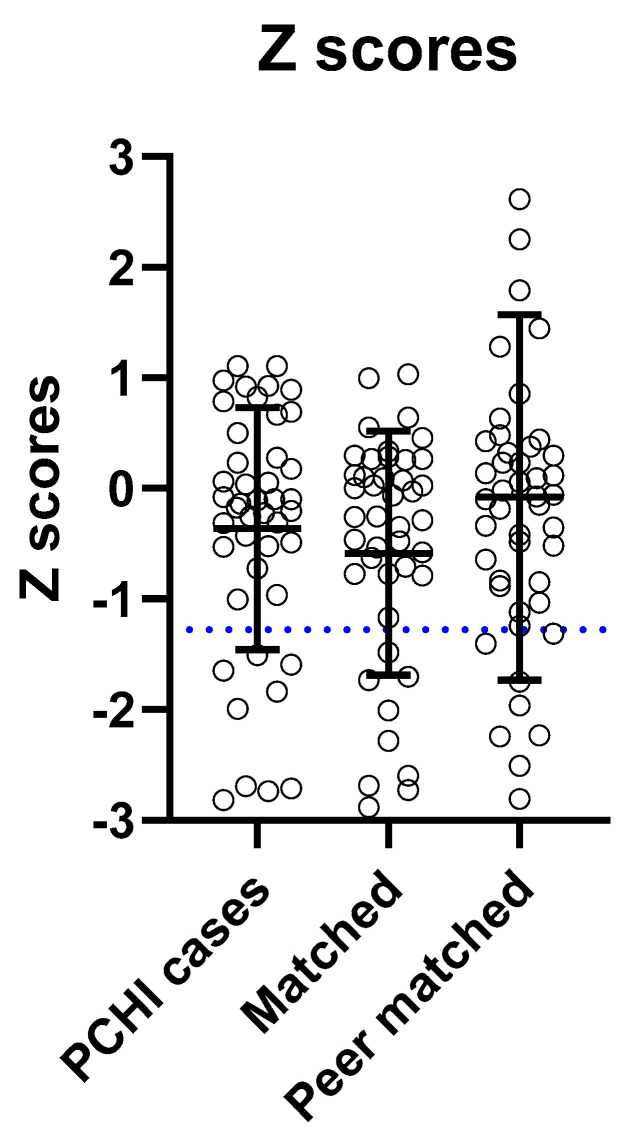
Birth weight z-scores were not significantly different between the groups. There were no statistically significant differences in the z-scores between groups (Kruskal–Wallis; *p* > 0.05). The horizontal bars represent the means, and the error bars the standard deviations. The blue dotted line indicates the z-scores of infants born small for gestational age (z-score < −1.28, 10th percentile) as defined by birth weight, gestational age, and sex.

We found no statistically significant differences between the z-scores (based on gestational age, weight, and sex) between cases with PCHI and peer-matched controls (Kruskal–Wallis; H = 1.50 (2); *p* = 0.47) (Figure 3). There was an even distribution of z-scores and most data for all groups were clustered around zero. It is evident from the boxplot that there is no visible difference in the variance between the parameters of the three groups. The peer-matched controls had a wider distribution of z-scores, as this cohort also included children who had z-scores > 1.28.

**Figure 4:**
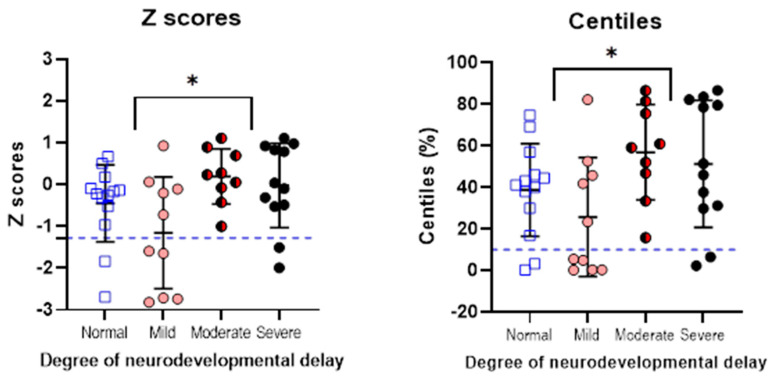
Z-scores and centiles for children with PCHI and different degrees of neurodevelopmental impairment. The two plots show the means (horizontal line) ±standard deviations (error bars) for the group with PCHI. Z-scores are shown on the left and centiles for birth weight, gestational age, and sex are shown on the right. The blue dotted line indicates the z-scores of infants born small for gestational age (z-score < −1.28, 10th percentile and centiles < 10%) as defined by birth weight, gestational age, and sex. * Indicates a level of significance of *p* < 0.05 when comparing the groups with normal/mild developmental delay to those with moderate/severe developmental delay for z-scores and centiles.

There was a significant difference in z-scores when normal-to-mild and moderate-to-severe neurodevelopmental outcomes were compared, per Mann–Whitney U test; U (44) = 347, *p* = 0.013. Paradoxically, children with normal-to-mild developmental impairment had lower z-scores than those with moderate-to-severe neurodevelopmental impairment (Figure 4). Similarly for centiles, those with normal-to-mild developmental outcomes had lower scoring centiles than those with moderate-to-severe developmental outcomes (Mann–Whitney U test; U (44) = 348, *p* = 0.012).

### 3.6. Neurodevelopmental Outcomes Are Significantly Poorer in Children with PCHI Compared with Matched Controls

Most children (70%) with PCHI were significantly more likely to have some degree of neurodevelopmental impairment than matched controls (16%) (Table 2). Importantly, impairments were not only restricted to speech and language (84% vs. 13%), as they were also more likely to have cerebral palsy (30% vs. 2%) and more often bilateral cerebral palsy than matched controls. Table 2 illustrates the subcategories of developmental impairments at 2 years for children with PCHI and the matched controls. Children with PCHI were significantly more likely to have moderate-to-severe developmental impairment (70%) than matched controls (16%) (Table 2). The odds ratio of a child having moderate-to-severe neurodevelopmental impairment if they had PCHI was 12.48 [95% CI = 3.37–46.40]; z = 3.77; *p* = 0.0002.

## 4. Discussion

The objectives of this study were to determine whether neurodevelopmental outcomes are significantly poorer in children with PCHI than matched controls (matched on gestational age, birth weight, and sex) and whether degree of prematurity is prognostic of PCHI and neurodevelopmental outcomes. In summary, we show that confirmed PCHI and NICU admission were associated with poor developmental outcomes at 2 years, and this was independent of prematurity.

Firstly, children with PCHI were more likely than matched controls to have severe neurodevelopmental impairment, not limited to speech and language difficulties. Secondly, children with PCHI were on average born more prematurely than the peer-matched controls. However, prematurity alone did not predict moderate-to-severe developmental outcomes at 2 years. The rate of moderate and severe neurodevelopmental impairment in the matched controls (7%) is in line with the reported rate of 4.3% from a meta-analysis of neurodevelopmental outcomes of preterm infants [3]. Hence, the PCHI NICU group studied represents an enriched population in which significant (moderate-to-severe) neurodevelopmental disability (47%) is greatly in excess of reported values (0.5% to 29%) [3].

Paradoxically, we show children with PCHI and a NICU admission with moderate-to-severe developmental delay had higher z-scores (clustering around the average) than those with normal-to-mild developmental delay. One explanation for this is that there was censorship for children with PCHI who were born more growth restricted (low z-scores), indicating many may not have survived to discharge from the NICU. These very sick children are unlikely to have had a hearing assessment, so we cannot know how many children fall into this category. 

Despite the low sensitivity of the ABR test used in the NHSP, it is the gold standard for identifying hearing loss in babies [37] and superior to using neonatal indicators for referral. Nevertheless, these data are important, since the NHSP is not available in all countries [38] and neonatal indicators and risk factors for PCHI are complex and likely compounding [10]. Therefore, understanding easily accessible neonatal indicators such as gestational age and birth weight could give us insight into possible mechanisms for surveillance. However, it should be noted that degree of prematurity can only be properly measured and assessed if there is reasonable access to public health and prenatal care.

A population-based study and systematic review (all children, not only those with NICU admission) found a 7% prevalence of moderate-to-profound hearing loss among children with cerebral palsy [39]. They also showed that nearly half of children with severe-to-profound sensorineural hearing loss had one or more additional medical problems [39]. In the current study, 70% of children with PCHI had some form of neurodevelopmental impairment and 30% had cerebral palsy, and most of these children (*n* = 11/13) had diplegic cerebral palsy. Cerebral palsy is closely associated with PCHI independent of prematurity, as only one child in the matched control group had cerebral palsy. One possibility is that there is a genetic cause which underlies both PCHI and cerebral palsy. However, it is also possible that PCHI is unrelated to the aetiology of cerebral palsy, since cerebral palsy and PCHI share many non-genetic risk factors (e.g., hypoxic–ischaemic encephalopathy, extracorporeal membrane oxygenation, neonatal hyperbilirubinemia, and neonatal meningitis) [17,18].

The connection between hearing deficits and cerebral palsy underscores the relationship between sensory impairments and motor disabilities. This emphasises the need for a multidisciplinary approach when it comes to addressing the diverse needs of this population.

Although the primary focus of this research was to assess the relationship between PCHI and prematurity with neurodevelopmental impairment we also examined the relationship between prematurity and PCHI. In a 2024 meta-analysis prematurity and small for gestational age (SGA) were not associated with PCHI [16]. Many studies had low or very low certainty of evidence. Associated factors with moderate-certainty evidence were low birth weight, congenital anomalies, craniofacial anomalies and intracranial haemorrhages [16]. Similarly, our data indicates that NICU infants with PCHI were overall not only restricted to the lower gestational ages and weights. When compared to peer controls they were ‘on average’ smaller and born at a more preterm age and had been in the NICU longer, however, the model (logistic regression) indicated that days in the NICU and not z-score were a significant predictor variable in the model for predicting PCHI. In accordance with the meta-analyses our analyses of z-scores indicated that there were no more infants with SGA (in the lowest 10% of z-scores/centiles) compared with matched or peer controls. A recent large population study (not included in the meta-analyses) found birth weight to be an independent risk factor for sensorineural hearing impairment. Intensive care treatments and comorbidities increased the risk, mainly for children born after 28 weeks gestation [40]. Thus, PCHI is complex and nuanced and likely related to associated factors—genetic vulnerability and environment in the NICU, with all that entails.

The main limitation in the current study is the potential for sampling bias. PCHI cases were only selected as those who had been referred from NHSP and then later confirmed to have a PCHI by an audiologist. Hence, babies with milder BHL could have been missed because of the current UK NHSP, which does not always detect milder hearing losses. Follow-up of acquired and progressive PCHI could be important to investigate factors which may mitigate against progression or target children who are susceptible. A further limitation was the single centre nature of the study. It will be important to replicate these analyses in other centres and in multi-centred studies.

## 5. Conclusions

Our findings support the notion that PCHI, along with NICU admission, significantly impacts neurodevelopmental outcomes. This underscores the importance of early identification and intervention for children with hearing impairments. The observation that developmental delays are not confined to speech and language suggests that PCHI and the associated NICU environment might influence broader neurodevelopmental pathways. This could include cognitive, motor, and behavioural domains. The data indicate that within this cohort, the degree of prematurity alone does not predict a more severe degree of neurodevelopmental delay. This could mean that the critical factor is the combination of PCHI and NICU admission rather than prematurity alone. This finding challenges the conventional focus solely on gestational age and birth weight and suggests a more nuanced approach to monitoring and intervention is needed.

Our findings suggest that any infant with PCHI and a history of NICU admission should be closely monitored, irrespective of their gestational age at birth. This may imply more comprehensive early intervention programs and follow-up strategies to mitigate potential developmental delays. Future research should seek to conduct longitudinal follow-up beyond 2 years to map the long-term developmental trajectory of these children and identify critical intervention points. Investigating the underlying mechanisms linking NICU admission, PCHI, and developmental delays could inform targeted interventions and therapeutic strategies. The impact of early hearing rehabilitation on developmental outcomes would be particularly important to study in this cohort.

## Figures and Tables

**Table 1:** Demographic data for PCHI cases, matched controls, and peer controls. Cases with PCHI were matched with controls on birth weight, gestational age, sex (21 female; matched controls 20 female, peer controls 24 female) and time in the NICU (days). Peer controls were matched on date of birth (±1 day) and sex. IQR = interquartile range (25 and 75% range). * Indicates statistically significant difference (*p* < 0.05) between cases and peer controls.

	PCHI Cases (*N* = 46)		Matched Controls (*N* = 46)		Peer Controls (*N* = 46)	
	Median	Range/ IQR	Median	Range/ IQR	Median	Range/ IQR
**Birth weight (g)**	1370	540–3420/ 960, 2648	1385	521–3650/ 955, 2653	2503 *	514–4220/ 1464, 3051
**Gestational age (weeks)**	32	24–41/ 28, 37	32	24–41/ 28, 37	36 *	25–42/ 31, 38
**Time on NICU (days)**	44	0–152/ 14, 100	25	1–140/ 7, 71	8 *	2–141/ 3, 31

**Table 2:** Neurodevelopmental outcome data at 2 years.

Developmental Outcome Data (*N* = 44)	PCHI	Matched Controls	Chi-Squared Value	*p* Value
Speech and language	26/44 (59%)	5/44 (11%)	22	<0.0001 *
Emotional-social difficulties	9/44 (20%)	4/44 (9%)	2	0.1 *
Cerebral palsy	13/44 (30%)	1/44 (2%)	12	0.0005 *
Bilateral cerebral palsy	11/44 (25%)	1/44 (2%)	10	0.002 *
Neurodevelopmental impairment (total)	31/44 (70%)	7/44 (16%)	23	**<0.00001**
**Degree of Neurodevelopmental Impairment (*N* = 44)**	**PCHI**	**Matched Controls**	**Chi-Squared Value**	***p* Value**
Mild	10/44 (23%)	4/44 (9%)	3	0.080 *
Moderate	9/44 (20%)	2/44 (5%)	5	0.02 *
Severe	12/44 (27%)	1/44 (2%)	11	0.001 *

Emboldened probability (*p*) values indicate a statistically significant difference between the cases (permanent childhood hearing impairment, PCHI) and matched controls (matched on gestational age, birth weight, and sex) using Chi-squared test, and for those groups where *n* ≤ 5 (in one or more groups, * Fischer’s exact was performed. *n* = 44 for cases with PCHI and matched controls. Two children with PCHI did not survive after discharge from the NICU. If there was no record of developmental delay or impairment in the notes, there was a presumption of normal outcomes. For speech and language outcomes, 26/31 (84%) children had problems with their speech and language compared to 5/39 (13%) matched controls.

## Data Availability

The original contributions presented in the study are included in the article, and further inquiries can be directed to the corresponding author.
